# PCR-reverse blot hybridization assay in respiratory specimens for rapid detection and differentiation of mycobacteria in HIV-negative population

**DOI:** 10.1186/s12879-021-05934-x

**Published:** 2021-03-16

**Authors:** Qing Zhang, Heping Xiao, Liping Yan

**Affiliations:** 1 Clinic and Research Center of Tuberculosis, Department of Tuberculosis, Shanghai, Key Laboratory of Tuberculosis, Shanghai Pulmonary Hospital, Tongji University School of Medicine, Shanghai, China; 2 Shanghai Clinic and Research Center of Tuberculosis, Department of Tuberculosis, Shanghai Key Laboratory of Tuberculosis, Shanghai Pulmonary Hospital, Tongji University School of Medicine, No. 507 Zhengmin Road, Shanghai, 200433 China

**Keywords:** *Mycobacterium tuberculosis* (MTB), Nontuberculous mycobacteria (NTM), Identification, PCR-reverse blot hybridization assay (PCR-REBA), Molecular diagnosis

## Abstract

**Background:**

Rapid identification of pathogenic *Mycobacterium* species is critical for a successful treatment. However, traditional method is time-consuming and cannot discriminate isolated non-tuberculosis mycobacteria (NTM) at species level. In the retrospective study, we evaluated the clinical applicability of PCR-reverse blot hybridization assay (PCR-REBA Myco-ID) with clinical specimens for rapid detection and differentiation of mycobacterial species.

**Methods:**

A total of 334 sputum and 362 bronchial alveolar lavage fluids (BALF) from 696 patients with mycobacterium pulmonary disease (MPD) and 210 patients with non-mycobacterium pulmonary disease used as controls were analyzed. Sputum or BALF were obtained for MGIT 960-TBc ID test and PCR-REBA Myco-ID assay. High resolution melt analysis (HRM) was used to resolve inconsistent results of MGIT 960-TBc ID test and PCR-REBA Myco-ID assay.

**Results:**

A total of 334 sputum and 362 BALF specimens from 696 MPD patients (292 MTB and 404 NTM) were eventually analyzed. In total, 292 MTBC and 436 NTM isolates (mixed infection of two species in 32 specimens) across 10 Mycobacterium species were identified. The most frequently isolated NTM species were *M. intracellulare* (*n* = 236, 54.1%), followed by *M. abscessus* (*n* = 106, 24.3%), *M. kansasii* (*n* = 46, 10.6%), *M. avium* (*n* = 36, 8.3%). Twenty-two cases had *M. intracellulare* and *M. abscessus* mixed infection and ten cases had *M. avium* and *M. abscessus* mixed infection. A high level of agreement (*n* = 696; 94.5%) was found between MGIT 960-TBc ID and PCR-REBA Myco-ID (*k* = 0.845, *P* = 0.000). PCR-REBA Myco-ID assay had higher AUC for both MTBC and NTM than MGIT 960-TBc ID test.

**Conclusion:**

PCR-REBA Myco-ID has the advantages of rapid, comparatively easy to perform, relatively low cost and superior accuracy in mycobacterial species identification compared with MGIT 960-TBc ID. We recommend it into workflow of mycobacterial laboratories especially in source-limited countries.

## Background

The genus *Mycobacterium* contains a number of acid-fast bacilli (AFB), including *Mycobacterium tuberculosis* complex (MTBC), *Mycobacterium leprae*, and non-tuberculosis mycobacteria (NTM) [1]. NTM are ubiquitous environmental organisms that incidentally cause opportunistic NTM pulmonary disease (NTM-PD) and extrapulmonary infections in immunocompromised individuals [2]. With the prevalence of acquired immunodeficiency syndrome (AIDS), the recognition of clinical importance of NTM is also growing [3]. Recently, many countries have reported a dramatically increased incidence of NTM-PD. [4–7] NTM infections constitutes 0.5–35% of all human mycobacterial infections [8]. For patients with risk factors, such as bronchiectasis, cystic fibrosis, and immune deficiency, the figure was higher still, over 50% [9]. However, a high percentage of patients have no risk factors [10]. Infections of most NTM species have been traditionally considered from environmental sources rather than person-to-person transmission like MTBC [11]. In addition, many NTM strains are insensitive to most anti-tuberculosis drugs [12, 13]. Therefore, quality and timely identification of mycobacterium species is necessary for better patient management and appropriate chemotherapy. However, most clinical features (cough, sputum production, fatigue, hemoptysis and fever) and abnormalities on chest radiography (centrilobular nodules, tree-in-bud opacity and cavitation) generally associated with NTM-PD are atypical, which may lead to false diagnoses as PTB, and hence to overtreatment of patients who do not have TB [14, 15]. The traditional methods used in mycobacterial laboratories require cultivated isolates and the results are only obtained after weeks to months of incubation. Therefore, these culture-based methods are time-consuming and labor-intensive [16]. Moreover, the traditional methods could not discriminate NTM to the species level, or detect mixed infections in which two or more mycobacterial species were simultaneously detected in the same specimen [17]. All NTM isolates from respiratory samples should be identified at least to species level, since that optimal therapeutic regimen differs according to different species, especially between slow-growing and rapid-growing species. Therefore, current clinical methods are not conducive to guide clinical decisions in a timely manner. Clinicians sometimes refer only to interferon-γ release assays (IGRAs). IGRAs are based on M. tuberculosis specific antigens. These antigens are not present in NTM strains other than *M. marinum, M. kansasii, M. gordonae and M. szulgai* [18]. However, it is worth mentioning that a number of TB patients are also negative in IGRAs [19]. So, IGRAs could not make a reliably distinction between NTM and MTBC.

Advances in molecular assays have accelerated the diagnosis of mycobacteria.

infections over recent years. Among these advances, nucleic acid amplification (NAA)-based techniques permit for identifying mycobacteria at the species level [20–23]. Sequencing of *16S rRNA* has been recommended as the gold standard method for definitive identification and discrimination of mycobacterial species [24]. Unfortunately, although fast, some of these techniques still require cultured strains and expensive equipment such as Hain line-probe and minion [22]. A commercial kit based on *16S rRNA* sequencing and nucleic acid probes and reverse blot hybridization (REBA), PCR-REBA Myco-ID (Yaneng BioSciences, Shenzhen), was developed for simultaneous genotyping of 22 clinical important mycobacterial species including MTB*, M. smegmatis, M. Intracellulare, M. kansasii, M. chelonae, M. marinum, M. fortuitum, M. terrae, M. nonchromogenicum, M. avium, M. scrofulaceum, M. abscessus, M. xenopi, M. gilvum, M. phlei, M. triviale, M. gordonae, M. gastri, M. vaccae, M. szulgai, M. diernhoferi, M. simiae*. Moreover, as a major advantage, PCR-REBA Myco-ID can be performed with clinical specimens. Despite the fact that this technology has been used as a rapid, low cost and easy-to-use method in mycobacterial research works, it has not been incorporated into workflow of many mycobacterial laboratories. Therefore, the purpose of this study was to evaluate clinical applicability of PCR-REBA Myco-ID assay for prompt and accurate identification of NTM at species level directly from 696 clinical specimens in China.

## Methods

### Ethics statement and informed consent

This study was approved by The Ethics Committee of the Shanghai Pulmonary Hospital, Tongji University School of Medicine in China (Approval number K20–422). Each participant gave written informed consent before enrollment. The study was performed according to the Declaration of Helsinki with respect to ethical principles for research involving application of human specimens.

### Clinical specimen collection

Clinically suspected NTM-PD patients admitted to Shanghai Pulmonary Hospital between January 3, 2018 and November 28, 2019 were eligible for screening if they met the following inclusion criteria: 1) HIV test negative; 2) providing two sputum specimens or bronchial alveolar lavage fluids (BALF) from sputum-scarce patients for MGIT 960-TBc ID test and PCR-REBA Myco-ID test. Samples should be processed within 24 h of collection.

### Fiberoptic bronchoscopy

Bronchoscopy procedures were performed as previously described [25].

### Routine identification methods

Nacetyl-L-cysteine (NALC)–NaOH method was used to decontaminate about 5 ml specimens [26]. The specimens were exposed to 4%NaOH for 15–20 min. The sediment was washed with sterile 0.9% NaCl solution and resuspended in 1.5 ml sterile 0.9% NaCl solution. Two separate 500-μl aliquots were prepared in 1.5 ml tubes for MGIT 960-TBc ID test and PCR-REBA Myco-ID test. Specimens were cultured by MGIT 960 (Becton Dickinson Diagnostic Systems, Sparks, MD) for 6 weeks following the standard procedure of the manufacturer [27]. In order to confirm the presence of mycobacteria and exclude contamination, samples from all positive MGIT 960 tubes were Ziehl-Neelsen (ZN) -stained and Gram-stained. TBc ID (Becton Dickinson, Sparks, MD) is an assay for the detection of MPT64 Ag, a mycobacterial protein secreted by MTBC and certain strains of *M. bovis*. 100-μl liquid media from the positive MGIT tubes was added to the TBc ID card and was incubated for 15 min at room temperature. The results were visually assessed. A positive test result indicated MTBC and a negative test result indicated NTM.

### PCR-reverse blot hybridization assay (PCR-REBA Myco-ID)

a) DNA Isolation: 500 μl bacterial precipitation was treated with DNA Lysis Buffer (10 mmol/L NaCl, 1 mg/ml SDS, 0.15 g/ml Chelex-1 s00 glass beads, 1% Tween 20) at 50 °C for 1 h, then at 100 °C for 10 min, and centrifuged at 10000 r/min for 2 min. The supernatant containing genomic DNA was transferred to another tube and preserved at − 20 °C for further PCR. b) PCR: PCR was carried out in a 50 μl reaction mixture including 5 μl DNA template, 10 mM Tris/HCl (pH 8.3), 50 mM KCl, 2 mM MgCl2, 0.2 mM dNTP, 0.4 mM each primer, and 2 U AmpliTaq Gold polymerase. First, the mixture was incubated at 94 °C, to activate the Taq polymerase, followed by 40 cycles of amplification (94 °C for 1 min, annealing and extension for 30s at 65 °C, 72 °C for 1 min), and finally incubated at 72 °C for 10 min. 5 μl PCR product was electrophoresed on 6% polyacrylamide gel with silver staining. c) REBA: The amplified PCR products were REBA tested with Mycobacterium species identification detecting Kit (Yaneng BioSciences, Shenzhen) according to the manufacturer’s guidelines. In brief, biotinylated PCR products were denatured at 25 °C for 5 min and were added to the REBA membrane strip in the blotting tray provided. Denatured single-stranded PCR products were hybridized with the probes on the strip at 55 °C for 30 min. The strips were then cleaned twice with gentle shaking in 1.0 ml of washing solution for 10 min at 55 °C, incubated at 25 °C for 30 min, and cleaned twice with 1.0 ml of CDS at room temperature for 1 min. Finally, visualize the signals of colorimetric hybridization and read the band pattern.

### PCR-high resolution melting (PCR-HRM) analysis

To confirm mycobacterium species inconsistently identified by the two different assays, fluorescence PCR-HRM Assay was carried out with Mycobacterium Identification Kit (Zeesan Biotech, Xiamen). The amplification of *rpoB* gene was performed on the following conditions: preincubation at 95 °C for 10 min, then denaturation at 95 °C for 10s, 45 cycles, annealing for 30s at 65 °C, and extension for 10s at 72 °C followed by the Tm analysis with increasing temperatures from 60 to 95 °C in a 0.2 °C s-1 slope increment for 10s. The HRM analysis was carried out with Gene Scanning Software Version 1.5.0 (Roche Instrument Centre, Switzerland). The aggregation of the melting curves was based on the regions of the melting curve corresponding to the pre-melting, melting, and post-melting regions. Distilled water was used as the non-template control (NTC).

### Statistical analysis

Data analysis was performed using SPSS for Windows (Version 19.0, SPSS Inc., Chicago). All patients were followed up for at least 6 months. The sensitivity, specificity, positive predictive value (PPV), and negative predictive value (NPV) of PCR-REBA Myco-ID assay and MGIT 960-TBc ID test was calculated. The categorical variables were analyzed using Fisher exact or Pearson *X*^*2*^ tests where appropriate and 2-tailed tests were used. The concordance of agreement between MGIT 960-TBc ID test and PCR-REBA Myco-ID assay was evaluated using Cohen’s kappa test (*k* > 0.75, excellent agreement; 0.4 < *k* < 0.75, moderate agreement; and *k* < 0.4, poor agreement). Receiver operating characteristic (ROC) curve analysis was performed and the area under curve (AUC) with a 95% confidence interval (CI) was further calculated. *P* < 0.05 was considered statistically significant.

## Results

### Demographic and clinical characteristics of the participants

**Fig. 1:**
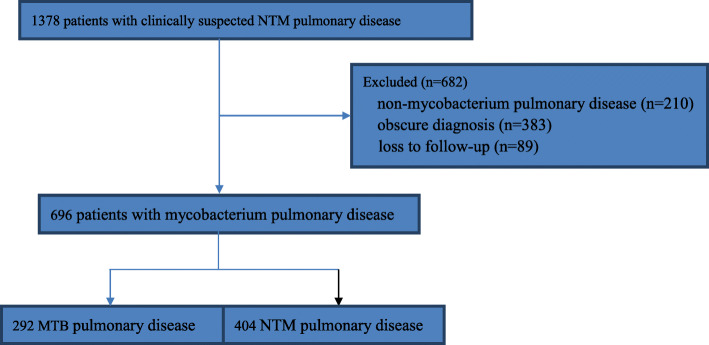
Flow chart of the study

In all, 1378 patients (698 sputum and 680 BALF) with clinically suspected NTM-PD were enrolled into this study. Finally, 682 patients were excluded, including 383 patients with obscure diagnosis, 89 patients lost to follow up and 210 patients with non-mycobacterium pulmonary disease (Table 1). The remaining 696 patients (334 sputum and 362 BALF) with mycobacterium pulmonary disease (MPD) (292 MTB and 404 NTM) were eventually analyzed (Fig. 1). Baseline characteristics of the 696 patients were summarized in Table 2. In total, 292 MTBC and 436 NTM isolates (mixed infection of two species in 32 specimens) across 10 Mycobacterium species were identified. The most frequently isolated NTM species were *M. intracellulare* (*n* = 236, 54.1%), followed by *M. abscessus* (*n* = 106, 24.3%), *M. kansasii* (*n* = 46, 10.6%), *M. avium* (*n* = 36, 8.3%), *M.s*crofulaceum (*n* = 4, 0.9%), *M. phlei* (*n* = 2, 0.5%), *M.*chelonae (*n* = 2, 0.5%), *M. xenopi* (*n* = 2, 0.5%), and *M. marinum* (*n* = 2, 0.5%). Twenty-two patients had *M. intracellulare* and *M. abscessus* mixed infection and ten patients had *M. avium* and *M. abscessus* mixed infection (Table 3).

**Table 1:** Baseline characteristics of patients with non-mycobacterium pulmonary disease

Condition	Age ± SD (range)	Gender (male: female)	MGIT 960 (+)	PCR-REBA (+)	Total (210)
Lung cancer	48 ± 16 (35–80)	49:38	0	0	87
Pneumonia	49 ± 17 (21–78)	35:30	1(NTM)	0	65
Pulmonary mycosis	51 ± 17 (35–80)	15:13	0	0	28
Silicosis	48 ± 8 (40–57)	20:1	0	0	21
Bronchogenic cyst	49 ± 4 (45–51)	1:1	0	0	2
Interstitial lung disease	50 ± 2 (48–52)	1:1	0	0	2
Pulmonary embolism	50 ± 1 (49–51)	1:0	1(MTBC)	0	1
Granulomatous vasculitis	43	0:1	0	0	1
Right lower lobe sequestration	46	1:0	0	0	1
Lung tissue X disease	51	1:0	0	0	1
Myelodysplastic syndrome	45	0:1	0	0	1

**Table 2:** Baseline characteristics of patients with mycobacterium pulmonary disease

	MTB pulmonary disease (*n* = 292)	NTM pulmonary disease (*n* = 404)
Age, SD (range)	44 ± 15 (25–80)	49 ± 17 (32–86)
Gender (male: female), n	190:102	191:213
Body mass index, median (range)	19.3 (14–29)	19.2 (13–28)
Diabetes mellitus, n	32	21
Autoimmune diseases, n	18	25
bronchiectasis or cystic fibrosis	101	98
Smear (+) / QFT-GIT^a^ (−)	73	240
No response to anti-TB medication	118	66

^a^QFT-GIT, QuantiFERON-TB Gold In-Tube

**Table 3:** Identification of clinical isolates using PCR-REBA

Clinical organism	Number of strains (728)	Identified by PCR-REBA (718)
MTB	292 (40.1%)	286 (97.9%)
*M. intracellulare*	236 (32.4%)	232 (98.3%)
*M. abscessus*	106 (14.6%)	106 (100%)
*M. kansasii*	46 (6.3%)	46 (100%)
*M. avium*	36 (4.9%)	36 (100%)
*M.scrofulaceum*	4 (0.5%)	4 (100%)
*M. xenopi*	2 (0.3%)	2 (100%)
*M. marinum*	2 (0.3%)	2 (100%)
*M. phlei*	2 (0.3%)	2 (100%)
M.chelonae	2 (0.3%)	2 (100%)

### Identification of mycobacterial by MGIT 960-TBc ID

Of the 292 pulmonary tuberculosis (PTB) patients, 274 isolates (93.8%) were identified as MTBC and 10 were negative in MGIT 960-TBc ID. Of the 404 NTM-PD patients, 394 isolates (96.5%) were identified as NTM and 6 were negative in MGIT 960-TBc ID (Table 4). Since there were two false-positive cases in the non-TB group, the specificity of MGIT 960-TBc ID test for both MTBC and NTM was 99.5%. Eight MTBC were misidentified as NTM, and four NTM misidentified as MTBC.

**Table 4:** Comparison of PCR-REBA and MGIT 960 for Detection of mycobacterium

		PCR-REBA	MGIT 960	*P*
MTB	sensitivity	97.9%	93.8%	0.012
	specificity	100%	99.5%	0.317
	PPV	100%	98.6%	0.040
	NPV	98.0%	96.7%	0.323
NTM	sensitivity	99.0%	96.5%	0.106
	specificity	100%	99.5%	0.317
	PPV	100%	98%	0.004
	NPV	99.0%	98.5%	0.530

### Identification of mycobacterial by PCR-REBA Myco-ID

Of the 292 PTB patients, 286 isolates (97.9%) were identified as MTBC and 6 were negative in PCR-REBA Myco-ID assay. Of the 404 NTM-PD patients, 400 isolates (99.0%) were identified as NTM and 4 were negative in PCR-REBA Myco-ID assay. The specificity and PPVs of PCR-REBA Myco-ID assay for both MTBC and NTM were 100%, which were significantly higher than those of MGIT 960-TBc ID (*P* = 0.04). The sensitivity of PCR-REBA Myco-ID to MTBC was also significantly higher than that of MGIT 960-TBc ID (*P* = 0.012).

### Consistency between MGIT 960-TBc ID and PCR-REBA Myco-ID

MGIT 960-TBc ID and PCR-REBA Myco-ID were concordant except for 38 samples (5.5%) (Table 5). A high level of agreement (*n* = 696; 94.5%) was found between MGIT 960-TBc ID and PCR-REBA Myco-ID (*k* = 0.845, *P* < 0.0001), indicating an optimal consistency between the 2 tests. For the 38 strains with inconsistent results of the two methods, PCR**-**HRM analysis was used for further analysis.

**Table 5:** Identification of clinical isolates using PCR-REBA, MGIT 960 and HRM

PCR-REBA	MGIT 960	HRM	Total (696)
MTB	(−)	MTB	10
MTB	NTM	MTB	8
(−)	MTB	MTB	6
*M.scrofulaceum*	MTB	*M.scrofulaceum*	4
(−)	NTM	*M. intracellular*	4
*M. intracellular*	(−)	*M. intracellular*	2
*M. phlei*	(−)	*M. phlei*	2
*M.chelonae*	(−)	*M.chelonae*	2
MTB	MTB	/	268
*M. intracellulare*	NTM	/	208
*M. abscessus*	NTM	/	74
*M. kansasii*	NTM	/	46
*M. avium*	NTM	/	26
*M. xenopi*	NTM	/	2
*M. marinum*	NTM	/	2
*M. intracellulare-M. abscessus*	NTM	/	22
*M. avium-M. abscessus*	NTM	/	10

### Establishment of ROC curve

**Fig. 2:**
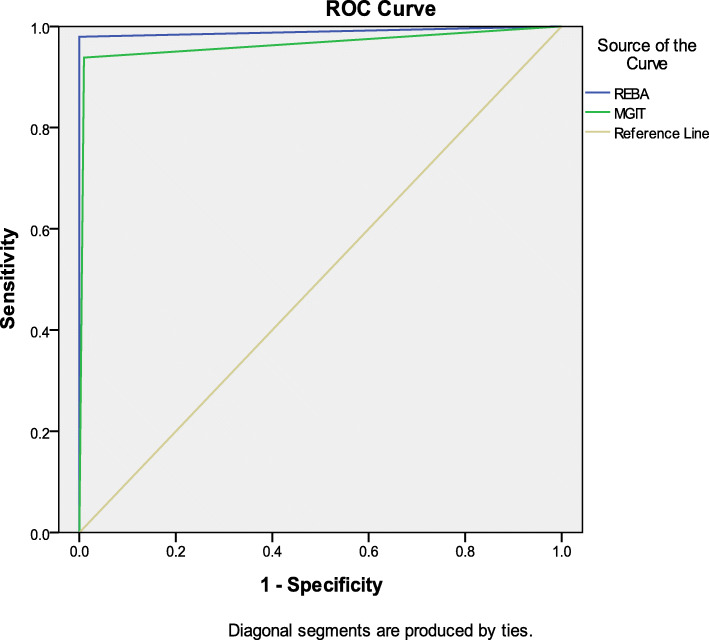
ROC curve of REBA and MGIT 960 for MTBC

**Fig. 3:**
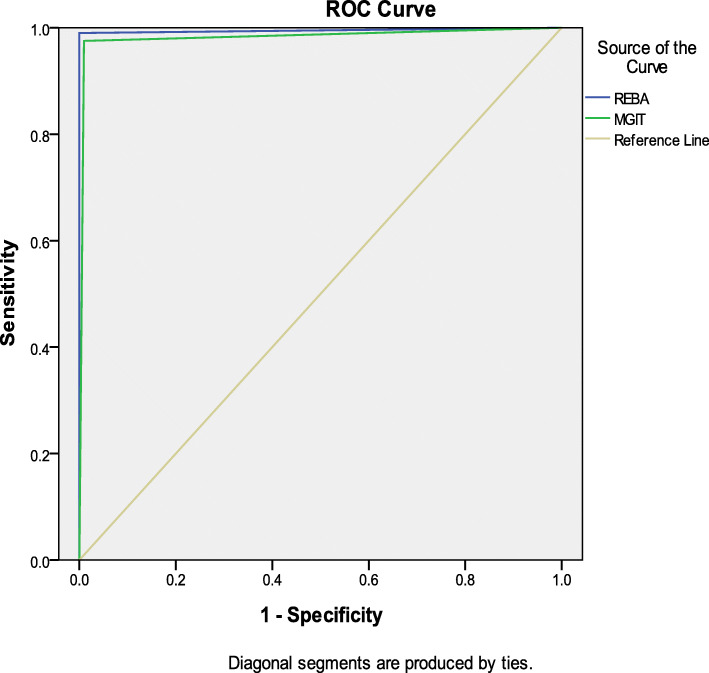
ROC curve of REBA and MGIT 960 for NTM

The AUC of PCR-REBA Myco-ID and MGIT 960-TBc ID for MTBC and NTM was 0.990 (95% *CI* 0.980–0.999) and 0.964 (95% *CI* 0.947–0.982), 0.995 (95% *CI* 0.988–1.0) and 0.983 (95% *CI* 0.971–0.994) respectively (Figs. 2, 3). PCR-REBA Myco-ID had the higher AUC for both MTBC and NTM than MGIT 960- TBc ID.

## Discussion

In current retrospective study, we evaluated the clinical applicability of PCR-REBA Myco-ID assay for rapid detection and differentiation of mycobacterial species directly from respiratory samples (sputum and BALF) in clinically suspected NTM-PD patients. Our results indicate that PCR-REBA Myco-ID assay has higher sensitivity and PPV than MGIT 960-TBc ID, which is consistent with the results of previous study by Lee et al. [28]. Because NTM are ubiquitous in the environment, a single isolated NTM strain from sputum without repeated isolation on culture, is not clinically significant, in general. Therefore, in the investigation of clinically suspected NTM-PD patients, sputum samples should be collected at least two other days for mycobacterium culture. Differentiation of *Mycobacteria* at the species level by phenotypic and biochemical testing is time-consuming because of the slow growth rate of mycobacteria. However, the major advantage of some direct molecular detection is to rapidly differentiate MTB from NTM in clinical samples. PCR-REBA Myco-ID assay has a short turnaround time of about 4 h. Timely intervention is also the key to the success of NTM treatment. Another limitation of MGIT 960-TBc ID test is that the accuracy is heavily affected by mycobacterial species and the quality of specimen. Relatively high contamination rate was reported [29]. Our results showed that the overall contamination rate was 1.7% (12/696) in this study. In addition, there were two false positive cultures in the non-TB group including one MTBC and one NTM isolate. Culture of NTM can be difficult even in *mycobacterial* reference laboratories. For example, while many NTM species grow best at 28 °C or 45 °C, conventional cultures are performed only at 35 °C. Other NTM species require prolonged incubation or special medium. Some NTM cultivars require supplementary media or extended culture cells. In our current study, six strains of NTM were not cultured, but were detected by PCR-REBA Myco-ID assay and confirmed by PCR-HRM analysis. Routinely culture also cannot discriminate NTM species. At present, more than 160 distinct *Mycobacterium* species have been validly published (http://www.bacterio.net/mycobacterium.html). The clinical relevance of isolated NTM differs strongly by species, from pathogenic species (e.g., *Mycobacterium kansasii* and *Mycobacterium malmoense*) to typical saprophytes (*Mycobacterium gordonae* and *Mycobacterium phlei)* [30]. In practice, treatment recommendations presented for NTM are based on the NTM species. NTM isolates species should be subspeciated. For example, the prognosis of diseases caused by *M. intracellulare* is better than that caused by *M. avium,* and the prognosis of diseases caused by *M. abscessus* is worse than that caused by *M. massiliense* [31, 32]. However, PCR-REBA Myco-ID assay cannot further classify *M. abscessus* as *M. massiliense*, *M. bolletii* and *M. abscessus* subspecies*.* If person-to-person transmission of *M. abscessus* is suspected, it is recommended to use whole genome sequencing (WGS), which has been developed in recent years [33]. Several methods based on WGS achieve subspecies-level resolution [34]. However, this technique is more sophisticated but not cost-effective and requires expensive equipment. It is too expensive to be used routinely in mycobacterial laboratories especially in source-limited countries. A limit of this study is that our method was not evaluated using metagenomic data. However, it should be noted that although we investigated 22 clinical important mycobacterial species in clinical samples, PCR-REBA Myco-ID assay appears to include the potential to analyze more NTM species in future studies.

Currently, mass spectrometry (MS) analysis is popular in large hospitals. For its low running costs. However, it was reported that closely related species could not be distinguished correctly [35]. Another limit of MS analysis is that this method still requires cultured NTM cells similar to Hain Geno Type MTBC test [36]. Polymerase chain reaction (PCR)-restriction fragment length polymorphism (RFLP) analysis (PRA) of some house-keeping genes does not need cultured NTM cells and special expensive equipment. However, PRA cannot discriminate different mycobacterial species in mixed infections [37]. In this study, 22 specimens with *M. intracellulare* and *M. abscessus* mixed infection and 10 specimens with *M. avium* and *M. abscessus* mixed infection were identified by PCR-REBA Myco-ID assay.

## Conclusion

Our finding showed that PCR-REBA Myco-ID assay is an efficient method and has higher specificity and rapidity than conventional methods. Furthermore, it does not require expensive specialized equipment. It should be incorporated into workflow of mycobacterial laboratories especially in source-limited countries.

## Data Availability

All data generated or analyzed during this study are included in this published article. The datasets used and/or analyzed during the current study are available from the corresponding author on reasonable request.

## Abbreviations

AFB: Acid-fast bacilli
AUC: Area under curve
BALF: Bronchial alveolar lavage fluids
HIV: Human immunodeficiency virus
HRM: High resolution melt analysis
MPD: Mycobacterium pulmonary disease
MTBC: *Mycobacterium tuberculosis* complex
NAATs: Nucleic acid amplification tests
NPV: Negative predictive value
NTM: Non- tuberculos mycobacteria
PCR-REBA: PCR-reverse blot hybridization assay
PPV: Positive predictive value
PTB: Pulmonary tuberculosis
ROC: Receiver operating characteristic
TB: Tuberculosis
IGRAs: Interferon-γ release assays
WHO: World Health Organization
